# The Risk of Therapy-Related Myelodysplasia/Acute Myeloid Leukemia in Hodgkin Lymphoma has Substantially Decreased in the ABVD Era Abolishing Mechlorethamine and Procarbazine and Limiting Volumes and Doses of Radiotherapy

**DOI:** 10.4084/MJHID.2012.022

**Published:** 2012-04-06

**Authors:** Ercole Brusamolino, Manuel Gotti, Valeria Fiaccadori

**Affiliations:** Clinica Ematologica, Department of Onco-Hematology, Fondazione IRCCS Policlinico San Matteo, University of Pavia, Pavia 27100, Italy

## Abstract

Patients with Hodgkin lymphoma treated with DNA-breaking alkylating agents such as mechlorethamine and procarbazine in the MOPP regimen and with topoisomerase II inhibitors, such as etoposide did show a long-term risk of developing therapy-related myelodysplasia and acute myelogenous leukaemia (MDS/AML). With the introduction of the ABVD (adriamycin, bleomycin, vinblastine, dacarbazine) regimen, this risk has substantially been reduced. In this review, different experiences are discussed to determine whether and how modifications of treatment in different cohorts of patients have reduced the overall risk of secondary MDS/AML. These data are drawn from large cohorts of patients treated over time with different therapies with an adequate follow-up.

## Introduction

Patients with Hodgkin lymphoma treated with DNA-breaking alkylating agents such as mechlorethamine and procarbazine in the MOPP regimen^1^ and with topoisomerase II inhibitors, such as etoposide did show a long-term risk of developing therapy-related myelodysplasia and acute myelogenous leukaemia (MDS/AML).^2^,^3^ With the introduction of the ABVD (adriamycin, bleomycin, vinblastine, dacarbazine) regimen, 4 this risk has substantially been reduced.^5^ In this review, different experiences are discussed to determine whether and how modifications of treatment in different cohorts of patients have reduced the overall risk of secondary MDS/AML. These data are drawn from large cohorts of patients treated over time with different therapies with an adequate follow-up.

## Historical data

In a large cooperative case-control study to assess the relative risk associated with several factors including age, splenectomy, combined modality therapy and cumulative dosage of alkylating agents and nitrosurea derivatives, we observed that the risk of leukemia in patients treated for Hodgkin lymphoma was significantly higher after combined modality programs than after chemotherapy alone (mostly the MOPP regimen) and was correlated with the extent of radiotherapy and type and duration of chemotherapy.^2^ **Table 1** illustrates the results of the logistic regression analysis in this series of 1659 patients followed for a median time of 10 years.^2^ Age over 40 years was not an independent risk variable for leukaemia, while a borderline significance was observed between increasing age and increased risk of subsequent leukaemia when the age was analyzed as a continuous variable. At variance, a significant influence was observed for advanced-stage versus early-stage disease (OR = 2.3; p = 0.03), for combined modality therapy (OR = 6.4; p = 0.02) and CT alone (OR = 4.1; p = 0.05) versus RT alone as reference group. In the combined modality therapy group, the MOPP combination + extended-field radiotherapy (EF-RT), either as adjuvant or salvage therapy, conferred a risk of leukemia 5.9 times higher than that given by chemotherapy without alkylating agents and procarbazine (p = 0.001). Among patients who received salvage therapy, the use of nitrosourea-containing regimens significantly increased the risk compared to that after MOPP or MOPP alternating to ABVD (OR = 8; p = 0.05).

**Figure 1.**
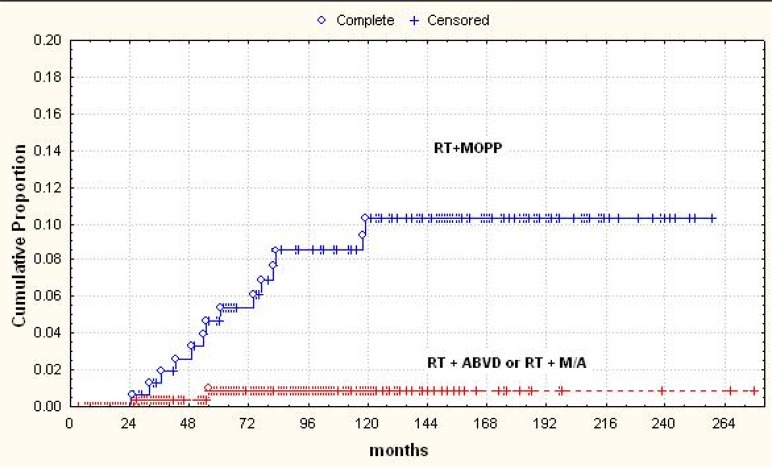
Risk of MDS/AML in Hodgkin lymphoma after combined modality treatment according to the type of chemotherapy.

Splenectomy was influential only in patients who had received MOPP chemotherapy (OR = 7.2; p = 0.002). A combined modality therapy including MOPP and RT (MOPP followed by adjuvant RT or RT followed by MOPP, or MOPP as salvage after RT) conveyed a higher risk of leukemia than alternating MOPP and ABVD + RT or ABVD alone + RT (p = 0.002; **Figure 1**). Likewise, in the 25-year experience of the National Cancer Institute of Milan, the cumulative risk of secondary MDS/AML after MOPP was 6.2% versus 1.7% in patients treated with ABVD.^6^ In conclusion, the “historical” risk factors for secondary MDS/AML in Hodgkin lymphoma were the use of mechlorethamine and procarbazine combined with EF-RT, splenectomy in patients receiving MOPP and salvage regimens containing nitrosurea derivatives.

## How the risk of MDS/AML has been reduced

We have modified over time our therapeutic approach in Hodgkin lymphoma reducing (with the alternating MOPP and ABVD regimens) and eventually abolishing mechlorethamine and procarbazine and limiting doses and volumes of radiotherapy. We analyzed three different cohorts of patients treated at our Institution from 1972 to 2003. The first cohort (A) includes 202 patients treated from 1972 to 1982 with MOPP ± RT; 46% of them received MOPP alone for advanced disease and 54% MOPP + EF-RT. The second cohort (B) includes 231 patients treated from 1983 to 1995 with alternated MOPP and ABVD (4+4 cycles) for advanced disease; 24% of them had been given RT. The third cohort (C) includes 207 patients treated from 1996 to 2003 (median FU: 12 yrs) with ABVD alone ± RT; 120 patients of this cohort (58%) received 4 cycles of ABVD and limited RT for early stage disease, while 42% received 8 cycles of ABVD and no RT for advanced-stage disease. Over a median follow-up of 12 years, the overall incidence of secondary MDS/AML was 2.6% (17 out of 640 total patients). **Table 2** illustrates the distribution of MDS/AML cases in the three different cohorts of treatment. The incidence of secondary MDS/AML in patients of the cohort A was 6% (12 out 202 patients), with a 10-year actuarial risk of 5.5%; most of the patients developing secondary leukemia in this cohort had received the combined modality treatment as a first-line treatment. In the cohort B, the crude rate of MDS/ASML was 2.6%, with 10-year actuarial risk of 2%; 5 out of 6 patients developing leukemia in this cohort had been given salvage therapy containing lomustine for relapse or resistant disease. No cases of secondary MDS/AML occurred in this cohort of patients that had been spared alkylating agents and procarbazine and given limited RT. A similar reduction of the leukemogenic risk according to therapy modifications over time has been registered in the Stanford University series.^7^ In this experience, three cohorts of patients have been analyzed. The entire first cohort of patients had received an alkylating-based therapy such as MOPP or PAVE (procarbazine, alkeran, vinblastine) and RT (extended-field RT in 73% of total); one fourth of patients in the second cohort had received an alkylating-based CT, alone (26%) or with RT (37%), while 74% had received a non alkylating-based CT such as VBM (vinblastine, bleomycin, methotrexate) or ABVD + IF-RT (37%). The large majority of patients in the third cohort (88% of total) had received the Stanford V regimen + RT. The incidence of MDS/AML was significantly lower (0.3%) in the third cohort of patients compared to the others (6.7 % in the first and 4.5% in the second cohort). The rarity of MDS/AML in the Stanford V regimen cohort indicates that limiting the cumulative dose of alkylating-based CT with lesser volumes of RT has significantly reduced the risk of subsequent leukemia.

In conclusion, abolishing mechlorethamine and splenectomy and substantially reducing procarbazine and radiotherapy has led to a consistent reduction of subsequent risk of leukemia in Hodgkin lymphoma.

We have obtained a confirmation of the very low leukemogenic risk related to the use of the ABVD combination from the analysis of our series of patients with clinical stage IA-IIA nonbulky Hodgkin lymphoma. No cases of secondary MDS/AML were observed among 120 patients treated with four cycles of ABVD + limited RT and followed for a median time of 15 years.^8^ Furthermore, no cases of MDS/AML were observed in the ABVD arm of a large Italian randomized trial comparing first-line ABVD x 8 cycles versus BEACOPP (4 escalated+4 standard) in advanced-stage Hodgkin lymphoma.^9^

## Risk of MDS/AML after autologous stem cells transplantation

An increased risk of secondary MDS/AML has been observed after salvage therapy with autologous bone marrow or peripheral stem cell transplantation in patients with resistant or relapsing Hodgkin lymphoma; the reported occurrence of this complication was quite variable in the different series.^10^–^13^ Besides, in a recent report,^14^ the comparison of conventional salvage therapy alone versus conventional therapy followed by autologous hematopoietic stem-cell transplantation did not show an increased risk of second neoplasms (including MDS/AML) after the high-dose procedure. In our experience, the 5-year actuarial risk of MDS/AML after autologous stem cell transplant in resistant or relapsing Hodgkin lymphoma was 2.9%, with a median latent time of 36 months from autologous transplant and a median 72 months from the time of the first exposure to cytotoxic therapy. Cytogenetic data in these cases were mostly consistent with a previous exposure to alkylating agents such chromosome 5 and or 7 alterations and/or deletions. In the GITMO (Gruppo Italiano Trapianto di Midollo Osseo) experience, the 5-yr actuarial risk of subsequent MDS/AML was 3.2%, with a latent time of 28 months. In this experience, significant protective factors were represented by shortening pre-tranplant debulking chemotherapy, avoiding TBI as conditioning regimen and rescuing with a large number of CD34+ cells, with a cut off at 8 × 10^6^/Kg. Taken together, the different data on the risk of subsequent MDS/AML after autologous stem cells transplant in Hodgkin lymphoma indicate the critical role of a pre-tranplant chemotherapy with alkylating agents and etoposide, while the high-dose procedure *per se* should not add to the risk when an appropriate number of progenitors cells is provided for the hematological recovery.

## Figures and Tables

**Table 1.** Logistic regression analysis of risk of leukemia in HL

**Variables**	**OR**	**95% CI**	**P value**
Age > 40 years	1.1	0.7–1.5	ns
Stage III–IV	2.3	1.1–5	0.03
Splenectomy	1.4	0.6–3.3	ns
MOPP alone	4.1	1.1–2.2	0.05
CT+RT	6.4	1.4–29	0.02
MOPP + EF-RT	5.9	2.3–12	0.001
MOPP + lomustine	8	4–25	0.05
MOPP and splenectomy	7.2	2.1–25	0.002

HL = Hodgkin lymphoma; ns = not statistically different

**Table 2:** Incidence of MDS/AML in Hodgkin lymphoma according to the era of treatment: The experience of the Hematological Clinic of Pavia.

**Time and Treatment**	**No. of pts (cohort)**	**Median FU (yrs)**	**No. MDS/AML**	**10-yr risk**
**1972–1983 MOPP ± RT**	202 (A)	20	12 (6%)	5.5%
**1984–1996 MOPP/ABVD ± RT**	231 (B)	16	6 (2.6%)	2%
**1990–2003 ABVD ± RT**	207 (C)	10	0	0
